# EP30 Sensory ganglionopathy as a possible presenting feature of Sjögren’s syndrome

**DOI:** 10.1093/rap/rkaa052.029

**Published:** 2020-11-03

**Authors:** Preeya Ummur, Kirsty Levasseur, Yee Suh Tee, Benjamin Fisher

**Affiliations:** University Hospitals Birmingham NHS Foundation Trust, Birmingham, United Kingdom

## Abstract

**Case report - Introduction:**

Primary Sjögren’s syndrome is a systemic autoimmune disease affecting the exocrine glands, commonly resulting in dryness of mouth and eyes. It can also rarely present with neurological symptoms most commonly peripheral neuropathies. The case highlights a rare case of sensory and motor neuropathy with some features suggestive of Sjögren’s syndrome as an underlying diagnosis.

**Case report - Case description:**

A 54-year-old Caucasian lady was transferred from a local hospital for specialist neurology care. She described widespread paraesthesia over two months progressing to lower limb weakness worsening over a two-day period. Her symptoms further progressed to involve her arms and new blurred vision. On further questioning she reported previous severe mouth ulcers, possible genital ulcers and painful jaw swelling with long standing history of dry mouth. On thorough neurological examination, the positive findings were mild ptosis of the right eye, paraesthesia on palpation of face, upper limbs had a flaccid tone with reduced power and pseudoathetosis of outstretched arms, reduced proprioception, and absent reflexes. Lower limbs were more affected with worse power and absent proprioception. Schirmer’s test showed normal tear production but saliva production was reduced.

MRI brain and spine were reported as pathological enhancement in both optic nerves, no obvious brain parenchymal abnormality, no obvious spinal cord abnormality. Lumbar puncture CSF pro 0.38, white cells 12 (poly 50%, mono 50%), red cells 3700, no organisms on gram stain, opening pressure 21.5 cmH_2_O. Sural nerve biopsy demonstrated profound loss of sensory axons, severe loss of large myelinated fibres in all fascicles with motor nerve involvement. Muscle biopsy demonstrated chronic neurogenic atrophy. Rheumatology screen showed positive anti Ro 52 antibodies (on an extended myositis screen). EMG demonstrated neurophysiological evidence of widespread marked loss of sensory axons in the upper and lower limbs and partial loss of motor axons in several lower limb territories. USS parotid glands showed some possibly mildly abnormal areas, minor salivary gland biopsy was normal. She received methylprednisolone, IV immunoglobulin and two courses of plasma exchange. Given some improvement following this treatment, further immunosuppression was felt to be appropriate. Initially Mycofenolate mofetil was started and Rituximab is now being considered.

**Case report - Discussion:**

This case demonstrates a mixed neuropathy picture. Following thorough investigations, many differential diagnoses of mixed neuropathy were considered and excluded (e.g. Guillain Barre, Syphilis, MS, HIV, Hepatitis). Neurological symptoms can develop before the onset of sicca symptoms in Sjögren’s syndrome. With a positive Anti Ro52, xerostomia and clinical improvement with methylprednisolone and plasmaphereses it seems likely there is an underlying autoimmune diagnosis in this case and despite a normal biopsy she would meet the 2016 ACR/EULAR classification criteria for Sjögren’s syndrome.

**Case report - Key learning points:**

Sjögren’s syndrome should be considered as a potential underlying cause in patients with a sensory ganglionopathy.

Neurological symptoms can develop before the onset of sicca symptoms in Sjögren’s syndrome.

